# Efficacy of medications in controlling cognitive dysfunction in Alzheimer’s : a systematic review

**DOI:** 10.1590/1980-5764-DN-2024-0243

**Published:** 2025-09-01

**Authors:** Sâmia Moreira de Andrade, Ítalo Sávio Mendes Rodrigues, Luís Marcelo Vieira Rosa, Rodrigo Luís Taminato, Gustavo Alves Andrade dos Santos

**Affiliations:** 1 Centro Universitário Santo Agostinho, São Luís MA, Brazil.; 2 Faculdade de Tecnologia de Teresina, Teresina PI, Brazil.; 3 Universidade Federal do Maranhão, São Luís MA, Brazil.; 4 Universidade Federal de Goiás, Goiânia GO, Brazil.; 5 Universidade de São Paulo, Faculdade de Medicina de Ribeirão Preto, Departamento de Anatomia e Cirurgia, Ribeirão Preto SP, Brazil.; 6 Faculdade São Leopoldo Mandic de Araras, Faculdade de Medicina, Departamento de Medicina, Araras SP, Brazil.; 7 Universidade Estadual de Campinas, Faculdade de Engenharia de Alimentos, Laboratórios de Nutrição e Metabolismo, Campinas SP, Brazil.

**Keywords:** Alzheimer Disease, Cognitive Dysfunction, Clinical Trial, Doença de Alzheimer, Disfunção Cognitiva, Ensaio Clínico

## Abstract

Dementia is one of the biggest global health crises of the 21st century. It is known that Alzheimer’s disease (AD) is the most common cause of dementia. Therefore, developing drugs with the potential to alter disease progression has been a priority.

**Objective:**

This study aims to review the efficacy and safety of drugs for AD.

**Methods:**

Based on the recommendations of the Preferred Reporting Items for Systematic Reviews and Meta-Analyses (PRISMA) document, a systematic review was carried out in different databases. The Alzheimer’s Disease Assessment Scale (ADAS-Cog) was used as the primary outcome. The risk of bias was checked using the Risk of Bias in Randomized Trials (RoB 2).

**Results:**

A systematic review resulted in 64 articles which were included in the analysis. The main medications discussed were: donepezil, which demonstrated a significant improvement in cognitive function, with good tolerability, although it has limitations in patients with swallowing difficulties. Transdermal patches showed similar effectiveness and were recently approved. Galantamine showed benefits on cognitive function, with good tolerability. Rivastigmine was effective and presented in transdermal form with similar efficacy to capsules, but had mainly gastrointestinal adverse effects. Pioglitazone showed no significant results initially, but some studies suggest benefits in patients with AD associated with type II diabetes mellitus.

**Conclusion:**

The studies reviewed indicate that, although some current medications such as donepezil, galantamine, and rivastigmine have modest, well-established benefits in clinical practice, many new agents have not yet demonstrated significant efficacy in modifying AD progression.

## RESUMO

A demência é uma das maiores crises de saúde globais do século XXI. Sabe-se que a doença de Alzheimer (DA) é a causa mais comum de demência, portanto o desenvolvimento de medicamentos com potencial para alterar a progressão da doença tem sido uma prioridade.

**Objetivo:**

Este estudo teve como objetivo revisar a eficácia e segurança de medicamentos para a DA.

**Métodos:**

Com base nas recomendações do documento Preferred Reporting Items for Systematic Reviews and Meta-Analyses (PRISMA), foi realizada uma revisão sistemática em diferentes bases de dados. A escala Alzheimer’s Disease Assessment Scale (ADAS-Cog) foi utilizada como desfecho primário. O risco de viés foi verificado utilizando o Risk of Bias in Randomized Trials (RoB 2).

**Resultados:**

Uma revisão sistemática resultou em 64 artigos, que foram incluídos na análise. Os principais medicamentos discutidos foram: donepezil, que demonstrou melhora significativa da função cognitiva, com boa tolerabilidade, embora apresente limitações em pacientes com dificuldades de deglutição. Os adesivos transdérmicos demonstraram eficácia semelhante e foram recentemente aprovados. A galantamina apresentou benefícios na função cognitiva, com boa tolerabilidade. A rivastigmina foi eficaz e apresentada na forma transdérmica com eficácia semelhante às cápsulas, mas com efeitos adversos principalmente gastrointestinais. A pioglitazona inicialmente não demonstrou resultados significativos, mas alguns estudos sugerem benefícios em pacientes com DA associada ao diabetes mellitus tipo II.

**Conclusão:**

Os estudos revisados demonstram que, embora alguns medicamentos atuais como o donepezila, a galantamina e a rivastigmina tenham benefícios modestos e bem estabelecidos na prática clínica, muitos novos agentes ainda não demonstraram eficácia significativa na modificação da progressão da DA.

## INTRODUCTION

Dementia is one of the biggest global health crises of the 21^st^ century. Currently, more than 50 million people live with dementia worldwide^1^. It is predicted that, by 2050, the prevalence of dementia will double in Europe and triple globally, with this estimate being three times higher when based on a biological (rather than clinical) definition of Alzheimer’s disease^2^. It is known that Alzheimer’s disease (AD) is the most common cause of dementia, responsible for 60–80% of cases, and is characterized by the deterioration of cognition, function and behavior, generally starting with memory loss of recent events. Elevated levels of β-amyloid (Aβ), which form extracellular senile plaques, and hyperphosphorylated tau (p-tau), which aggregates intracellularly as neurofibrillary tangles, are found in the brain tissues of AD patients, these being the main pathological characteristics^1,3^.

Although the symptoms present in AD can be temporarily alleviated by detailed care and medication, there are no specific measures to prevent or cure Alzheimer’s disease^4^. Current therapeutic agents for dementia related to Alzheimer’s disease temporarily improve symptoms but do not alter the course of the underlying disease. Interventions aimed at removing amyloid plaques are believed to slow the clinical progression of Alzheimer’s disease. Positively, an anti-amyloid antibody (aducanumab) has received accelerated approval from the Food and Drug Administration^5^.

Still, current AD treatment with donepezil, galantamine, rivastigmine, and memantine is symptomatic only and offers modest benefits. Therefore, the development of drugs with the potential to alter disease progression has been a priority. An increasing number of clinical trials have emerged to test the efficacy of potential therapies aimed at treating AD, with β-amyloid having been the main focus for more than 30 years. However, highly promising medicines have recently failed to demonstrate clinical benefits in phase III trials, making it necessary for these data to be synthesized and disseminated^6^. Therefore, in the present review, we will discuss the efficacy in relation to cognitive dysfunction as well as the safety of potential agents tested in clinical trials in Alzheimer’s disease.

## METHODS

### Protocol

The protocol used followed the recommendations of the Preferred Reporting Items for Systematic Reviews and Meta-Analyses (PRISMA) document^7^.

### Eligibility criteria

The guiding question was: “What is the efficacy and safety profile of medications used or under investigation for cognitive dysfunction in Alzheimer’s disease?” For this, the acronym PICO was used: Population (Patients with Alzheimer’s), Intervention (therapeutic intervention or maintenance of therapy), Comparison or Control (between therapies used or control group), and Outcomes (stabilization or delay in the decline of cognition, function and behavior).

Randomized or non-randomized clinical trials carried out with patients with or probably diagnosed with Alzheimer’s disease were eligible, following the fourth edition of the Mental Disorders Diagnostics and Statistics Manual (DSM-IV) and the National Association of Nervous and Communicative Disorders and Stroke/ Alzheimer’s Disease Institute of Standards (NINCDS)^8^. Complete articles published in English were included, without time limits.

Exclusion criteria: duplicate articles in the databases; review articles, meeting summaries or case reports/series; non-human studies; studies without specification of the therapeutic regimen and studies that did not use the Cognitive Subscale of the Alzheimer’s Disease Assessment Scale-Cognitive Subscale (ADAS-Cog) to assess the effectiveness of treatment.

### Sources of information

The databases used were Medical Literature Analysis and Retrieval System Online—Medline (via the United States National Library of Medicine—PubMed), Latin American and Caribbean Health Sciences Literature (Lilacs), Scientific Electronic Library Online (SciELO), Excerpta Medica Database (EMBASE), Cochrane Central Register of Controlled Trials (CENTRAL) and Google Scholar. All references were selected until February 17, 2024.

### Paper search

The search strategy was adapted for each database, with the descriptors chosen through the list of Health Sciences Descriptors (DeCS, for Lilacs), Medical Subject Headings (Mesh, for Medline and CENTRAL) and EMTREE (for EMBASE); LILACS: Alzheimer’s Disease AND clinical trial; PubMed: (((Alzheimer’s Disease) AND (Clinical trial)) AND (efficiency)) NOT (review); CENTRAL: “Alzheimer’s disease” AND “clinical trial”; EMBASE: alzheimer AND (‘disease’/exp OR disease) AND ‘therapy’ AND ‘clinical trial’; SciELO: ((Alzheimer Disease) AND (treatment) OR (therapeutic)).

### Study selection

Two researchers independently selected the articles, with discrepancies eliminated by a third reviewer. With the help of the Rayyan platform (http://rayyan.qcri.org), titles and abstracts were evaluated, in which previously selected studies of interest were read in full to confirm their inclusion.

### Data collection process

Data extraction was performed by three reviewers. The variables described were: year of publication, study design, treatment regimen, duration of follow-up, efficacy, safety, side effects and comparator.

To assess efficacy, the Cognitive Subscale of the Alzheimer’s Disease Assessment Scale (ADAS-Cog) was adopted as the primary clinical outcome. It is one of the most commonly used cognitive scales in clinical trials and is considered the ‘gold standard’ for evaluating anti-dementia treatments^9^.

### Bias risk assessment

Two independent reviewers assessed the risk of bias using the Risk of Bias in Randomized Trials (RoB 2), a tool for analyzing randomized studies^10^. For non-randomized studies, the tool used was the Risk of Bias in Non-Randomized Studies of Interventions (ROBINS-I)^11^. The risk of bias graphs was created using the robvis tool^12^.

## RESULTS

**Figure 1:**
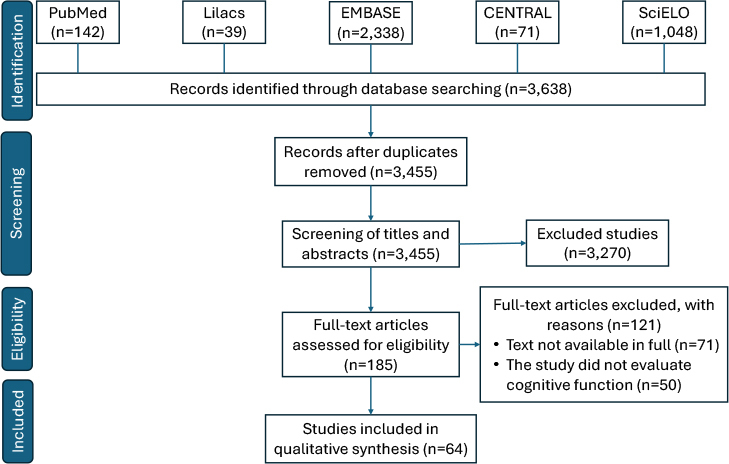
Article selection flowchart (papers).

The search in the databases resulted in 3,638 articles (Figure 1). Initially, 183 duplicate articles were excluded from the databases. After screening, 185 articles were selected for full reading, of which 64 were included in the qualitative analysis.

**Figure 2:**
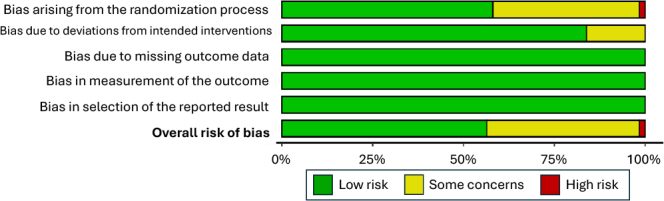
General assessment of the risk of bias of the studies.

Therefore, the importance of synthesizing the results found so far to guide new investigations stands out. Figure 2 shows the result of the risk of bias assessment, where it is possible to observe that the majority of selected studies presented a low risk of bias, indicating that they present good quality of the evidence presented, indicating that the analysis and conclusions are less likely to be distorted by systematic errors or external influences.

Table 1 exemplifies the medications reported in different Randomized Clinical Trials for mild to moderate Alzheimer’s Disease, which will be discussed in specific topics in this review. Among the 68 medications, the most evaluated were donepezil (n=8), galantamine (n=5) and rivastigmine (n=3), which are medications already used in clinical practice for the treatment of Alzheimer’s^13^. A direct comparison between these medications is also reported in Table 2 to facilitate comparison between them.

**Table 1:** Medicines evaluated in the present study.

Medicine	Number of publications
Donepezil	7
Galantamine	5
Rivastigmine	3
Pioglitazone	2
Doxycycline	2
Rifampicin	2
Resveratrol	2
Metrifonate	2
Acetyl-L-carnitine (ALCAR)	1
Saffron	1
Tulrampator (S47445)	1
Fisostigmine	1
Cerebrolysin	1
Tacrine	1
Eptastigmine	1
Diclofenac	1
Misoprostol	1
PF-05212377 (SAM-760)	1
Scyllo-Inositol (ELND005)	1
Semagacestat	1
Huperzine A	1
Tideglusibe	1
Sodium selenate	1
Capric/Caprylic Acid Triglycerides	1
Vitamin D2	1
ABT-288	1
Simvastatin	1
Atomoxetine	1
Benfotiamine	1
Docosahexaenoic acid	1
Bexarotene	1
Hydroxychloroquine	1
Levetiracetam	1
Masupirdine	1
Rotigotine	1
Tarenflurbil	1
NeuroAiD™ (MLC601)	1
Octohydroaminoacridine	1
Velnacrine	1
EHT0202 (Etazolate hydrochloride)	1
Vitamin B	1
MK-7622	1
Edonerpic maleate (T-817MA)	1
Idalopirdine	1
UB-311	1
SB-742457	1
Omega-3	1
Tramiprosate	1
Velnacrine	1
Blarcamesine	1
Alphoscerate	1
Posiphen	1

**Table 2:** Pharmacological comparison between the most cited medications in the literature*.

Drug	Pharmacological class	Starting dose and maximum dose	Route of administration	Efficiency
Donepezil	Acetylcholinesterase (iAChE) inhibitors	Starting dose:5 mg once a day (at night, before bed).Maximum dose:10 mg once a day (for mild to moderate Alzheimer’s).23 mg once daily (for moderate to severe Alzheimer’s, in patients who have tolerated 10 mg for at least 3 months).	Oral	Donepezil has demonstrated modest improvements in cognition in patients with mild to moderate Alzheimer’s disease. Its effect is symptomatic, that is, it temporarily improves cognitive functions, but does not stop the progression of the disease.
Rivastigmine	Acetylcholinesterase (iAChE) inhibitors and butyrylcholinesterase (BuChE) inhibitors	Oral route (capsules or oral solution)Starting dose: 1.5 mg twice a dayMaximum dose: 6 mg twice a day (12 mg/day) Transdermal route (skin patch)Initial dose: 4.6 mg/24h (1 patch per day)Maximum dose: 13.3 mg/24h (for patients who tolerate 9.5 mg/24h for at least 4 weeks).	Oral or transdermal	Rivastigmine has proven efficacy in the treatment of mild to moderate Alzheimer’s disease and dementia associated with Parkinson’s disease. Its effect is symptomatic, that is, it temporarily improves cognition and functionality, but does not stop the progression of the disease.
Galantamine	Acetylcholinesterase inhibitors and nicotinic allosteric modulators	Oral route (capsules or oral solution)Starting dose: 4 mg twice a dayMaximum dose: 12 mg twice a day (24 mg/day) Transdermal route (skin patch)Initial dose: 5 mg/24h (1 patch per day)Maximum dose: 10 mg/24h	Oral or transdermal	Galantamine is effective in treating mild to moderate Alzheimer’s disease, improving cognition, functionality and neuropsychiatric symptoms. Its dual mechanism (acetylcholinesterase inhibition + nicotinic modulation) provides additional benefits over other acetylcholinesterase inhibitors.

Note: *This table addresses a comparison of cognitive effectiveness in Alzheimer’s disease between medications commonly indicated for the disease.

## DISCUSSION

The studies evaluated were published between 1994 and 2025, demonstrating that clinical research on Alzheimer’s disease is an important topic that continues to this day. In fact, Alzheimer’s disease is the most common cause of dementia, which is considered one of the biggest global health crises of the 21^st^ century^1^.

Recognition of the entire pathophysiological process involved in the onset and progress of AD should serve as the basis for the pharmacological treatment of Alzheimer’s disease. In this sense, many hypotheses about the causes and possible treatments have been established, some have already been discarded and others are in the confirmation phase. It is believed that the entire neurodegenerative process is triggered by a series of reactions involving neurotransmitters, proteins, and other molecules, some of which were previously unknown. Some elements such as lifestyle, age and comorbidities, in addition to genetic aspects, can make meaning, as long as there is some type of relationship with the disease^14^. Below, we will discuss each medication found in this review individually and in detail.

### Donepezil

Donepezil is a centrally acting, rapid and reversible acetylcholinesterase inhibitor. By binding to acetylcholinesterase, it inhibits the hydrolysis of acetylcholine, thus increasing the availability of this neurotransmitter in synapses and improving cholinergic transmission^15^. The FDA approved its marketing, currently the most used medication in the treatment of dementia and cognitive symptoms in Alzheimer’s disease^16^.

The first Randomized Clinical Trials (RCTs), carried out by Rogers and colleagues in 1996 and 1998^17,18^, demonstrated that cognitive function, based on the ADAS-Cog scale, significantly improved in the 5 and 10 mg/day donepezil groups compared with the placebo in the initial 12 weeks and extended up to 24 weeks. Other clinical trials confirmed this benefit, demonstrating that this medication is effective and well tolerated^19-21^. Despite being effective, this oral medication has some limitations such as difficulty in administration when the patient is unable to swallow^22^. Therefore, it becomes necessary to develop new pharmaceutical formulations to deliver the medicine to the patient. To this end, transdermal donepezil IPI-30 patches (87.5 mg/25 cm2 of IPI-301) were tested, demonstrating similar effects to the 5 mg donepezil tablet on patients’ cognitive function after 24 weeks. The use of patches was associated with a greater number of adverse events, however mild, such as pruritus or erythema, which demonstrates safety and efficacy for patients with mild to moderate AD when compared to oral donepezil^23^. Interestingly, in relation to cognition, donepezil demonstrated a better effect when compared to galantamine, with both drugs being well tolerated, although galantamine presented more gastrointestinal adverse events than donepezil^24^. Positively, in March 2022, the FDA approved the first transdermal system for the administration of donepezil in patients with mild, moderate and severe AD^25^.

Post-treatment adverse events include: nausea, diarrhea, anorexia, vomiting, associated with cholinergic side effects, as well as headache, urinary tract infection, dizziness, agitation, insomnia, confusion, depression and anxiety^17-19^. Still, studies show that donepezil has good safety, as well as efficacy.

### Galantamine

In RCTs, they demonstrated that galantamine presented significant benefits on cognitive function at doses of 16, 18, 24 and 36 mg/day^26,27^. The dose of 4 mg twice daily during weeks 1–4 and 8 mg twice daily during weeks 5–13 also showed positive results with safety and tolerability comparable to donepezil^28^. The study by Wilcock et al.^28^ reported superior efficacy of galantamine in maintaining cognition and similar safety and tolerability compared to donepezil, suggesting that galantamine should be considered in the initial and long-term treatment of patients with mild to moderate Alzheimer’s disease. In fact, this drug, like donepezil, acts by inhibiting acetylcholinesterase. Additionally, galantamine exerts a positive modulation on nicotinic acetylcholine receptors, which can enhance the release of this neurotransmitter^29^.

Slow dose escalation appears to improve the tolerability of galantamine, minimizing the incidence and severity of adverse events. The incidence of adverse events in the galantamine groups presented as gastrointestinal symptoms was low and the majority of adverse events were mild, such as nausea (13% [33/261]), vomiting (6% [15/261]), dizziness (5% [13/261]) and anorexia (4% [11/261])^26,27^.

### Rivastigmine

Rivastigmine also acts as an inhibitor of the enzyme acetylcholinesterase, in addition to inhibiting butyrylcholinesterase. Since these enzymes are responsible for the degradation of acetylcholine, their use guarantees a greater concentration of this neurotransmitter in the synapse, thus improving communication between neurons and consequently cognition^30^.

RCTs demonstrate that rivastigmine provides benefits at doses of 1–4 mg/day with visible results in the first six months, which increased after 12 months. Increasing to higher doses (6–12 mg/day) was not associated with an increase in mortality risk^31,32^. This medication was also evaluated with another pharmaceutical formulation, where patients received a patch with 9.5 mg/24 h (10 cm^2^) of rivastigmine once a day compared to a capsule twice a day (12 mg/day). Positively, considering the efficacy parameters under consideration, both treatments demonstrated similar performance at Week 24^33^. Rivastigmine in patch form significantly reduces the levels of the enzyme butyrylcholinesterase, closely related to the reduction of acetylcholine in the synaptic cleft, when compared to the oral form, demonstrating the advantages of transdermal use, in addition to reducing the risk of drug interactions^34^.

The most common adverse symptoms were gastrointestinal, e.g. nausea, vomiting, diarrhea and anorexia. Other less frequent adverse events included confusion and agitation, decreased appetite, dizziness, weight loss, hypertension and itching at the application site^31-33^.

### Pioglitazone

Pioglitazone is an efficient PPARγ agonist, thus presenting anti-inflammatory properties that can prevent problems with cognitive deterioration^35^. Individuals with mild-moderate Alzheimer’s who received 15 mg pioglitazone tablets or placebo per day, increased from one tablet per week to three tablets per day to reach a maximum dose of 45 mg pioglitazone/day showed no improvement on the ADAS-Cog scale or any other clinical outcome. The researchers did not rule out the possibility that the lack of a statistically significant treatment effect was related to the small sample size. Interestingly, even though this is a medication for the treatment of diabetes, its administration for 18 months did not promote significant changes in blood glucose levels even in non-diabetic individuals^36^. On the other hand, another clinical trial, this time carried out in patients with AD associated with type II diabetes mellitus, demonstrated that its daily use at a dose of 15–30 mg was sufficient to promote cognitive and functional improvements and stabilization of the disease in diabetic patients with AD^37^. The limited number of RCTs makes it difficult to reach a broader conclusion, especially because there is a divergence in the results found in the studies. However, to date, we cannot rule out the possibility of a positive effect of pioglitazone on the cognitive function of patients with AD, and future investigations are needed to better explore its effects. Its most common adverse effect was edema, which is already a widely recognized effect for this medication. Still, this adverse effect was tolerated without discontinuation of study medication^36,37^.

### Doxycycline and rifampicin

Doxycycline was evaluated in combination with rifampicin. While doxycycline inhibits the translation of messenger RNA (mRNA) into proteins, rifampicin acts to inhibit bacterial RNA polymerase^38,39^. Although these drugs have long been used as antibiotics, recently, studies have shown that they are neuroprotective in models of neurodegenerative diseases and brain injuries, mainly due to their anti-inflammatory and antiapoptotic effects^40,41^. Patients with mild-moderate AD received daily oral doses of doxycycline 200 mg and rifampicin 300 mg or placebo for three months. Positively, this combination showed a smaller decline in ADAS-cog score at six months when compared to the placebo group, without showing a statistically significant difference in the percentages of patients who presented adverse events^42^. However, another study carried out over a 12-month period of treatment with doxycycline 100 mg twice a day + rifampicin 300 mg a day did not demonstrate statistical significance in the decline/deterioration of cognitive function in patients with AD compared to placebo^43^. Therefore, conflicting data prevents a conclusion about its effectiveness, requiring further future investigations. Adverse effects were: anorexia, sleep disturbances, nausea, weight loss and diarrhea^42,43^.

### Resveratrol

Resveratrol is marketed as a phytonutrient and is considered neuroprotective^44^. Patients with AD who received placebo or resveratrol 500 mg orally once a day (with dose escalation in 500 mg increments every 13 weeks, ending with 1,000 mg twice daily) showed no improvement or delay in decline in function cognitive^45^. Furthermore, the use of 5 mg of resveratrol per dose administered twice a day in liquid form also did not reach statistical significance in relation to this parameter^46^. These studies indicate that resveratrol is not capable of slowing cognitive decline in patients with AD. Despite this, its use is safe and well tolerated, without adverse effects^45,46^.

### Metrifonate

Metrifonate is also an inhibitor of the acetylcholinesterase enzyme, and is currently marketed as an anti-parasitic^47^. Monitoring patients with AD for 18 months using metrifonate (5 mg/kg of body weight for two weeks and 4.9 mg/kg for one week) revealed that this medication was able to reduce the decline in cognition^48^. This fact was confirmed in a subsequent study using the following treatment regimens:

- dose of 40/50 mg of metrifonate: 40 mg in patients weighing less than 65 kg and 50 mg in patients weighing 65 kg (i.e. equivalent to approximately 0.65 mg/kg/day);
- 60/80 mg dose of metrifonate: 60 mg in patients weighing 565 kg and 80 mg in patients weighing 565 kg (i.e., equivalent to approximately 1.0 mg/kg/day);
- placebo.

In this case, ADAS-cog was significantly improved in the 60/80 mg and 40/50 mg dose groups, compared to placebo^49^.

Its reported adverse effects were: dizziness, restlessness, slight and gradual decrease in hemoglobin, hematocrit and red blood cell count, chest discomfort, atrial fibrillation, headache and hiccups. However, these events were rare and did not require adjustment of the metrifonate dose or discontinuation of treatment^48,49^.

### Medications with potential use in Alzheimer’s disease

Analysis of the listed medications and their clinical studies can provide insight into the development of treatments for AD and the potential impact of these medications on delaying cognitive decline. Among these medicines, the following stand out:

- Physostigmine^50^: Acetylcholinesterase inhibitor, which promotes the increase in acetylcholine, as well as donepezil;
- Cerebrolysin^51^: It is a mixture of peptides and amino acids with neuroprotective properties;
- Tacrine^52^: It was one of the first acetylcholinesterase inhibitors approved for the treatment of AD. Although it has shown modest benefits, tacrine has been taken off the market in many places due to its hepatic side effects;
- Eptastigmine^53^: This medication is also an acetylcholinesterase inhibitor;
- Benfotiamine^54^: It is a synthetic form of vitamin B1 (thiamine) that has been studied for its neuroprotective potential. Evidence on its effectiveness in treating AD is still limited, and more studies are needed to determine its impact;
- Rotigotine^55^: It is a dopamine agonist used mainly for Parkinson’s disease;
- Octohydroaminoacridine^56^: A compound with acetylcholinesterase inhibitory properties, with initial studies indicating possible benefits in AD. However, more research is needed to confirm its effectiveness;
- Idalopirdine^57^: An inhibitor of acetylcholinesterase and the serotonin receptor. Studies have shown a modest effect on cognitive function, but the evidence is not enough for a clear consensus;
- SB-742457^20^: It is an acetylcholinesterase inhibitor with distinct properties, but available data is limited and does not support a significant benefit compared to established treatments;
- Velnacrine^58^: An acetylcholinesterase inhibitor that has had some initial success, but clinical use has been limited due to concerns about side effects and the availability of safer, more effective alternatives;
- Blarcamesine^59^: SIGMAR1 (sigma non-opioid intracellular receptor 1) agonists in the central nervous system (CNS). It showed delayed clinical progression by 49.8% at 48 weeks on the pre-specified ADAS-Cog primary cognitive endpoint, with no side effects;
- Alphoscerate^60^: This is a phospholipid containing choline, which is a precursor to acetylcholine. This treatment has been shown to be safe and effective in improving cognitive function in study subjects with mild cognitive impairment, but further studies are needed to consolidate this research.

In summary, many of these medications have shown some initial evidence of potential benefit in the treatment of AD, but the limited number of studies and variability in results make it difficult to draw definitive conclusions. Research remains essential to determine which treatments are most effective and safe for slowing the cognitive decline associated with AD.

### Other medications that have not been shown to be effective in reducing cognitive decline in Alzheimer’s disease

The development of treatments for AD is an active field of research, and many medications and supplements have been studied with the hope of slowing cognitive decline. However, some of these treatments have not demonstrated significant effectiveness in clinical trials. Among these medications and supplements that did not present clear benefits in reducing cognitive decline in AD, the following stand out:

- Acetyl-L-carnitine (ALCAR)^61^: This supplement has been studied for its neuroprotective properties and potential cognitive effect. However, the results were inconclusive, and there was insufficient evidence to support its use as an effective treatment for AD;
- Saffron^62^: Studies on saffron (Crocus sativus) have shown mixed results. Although some initial research suggested a potential benefit, the overall evidence was not sufficient to recommend its clinical use;
- Tulrampator (S47445)^63^: An AMPA receptor modulator studied for AD. Studies have not shown significant benefits in terms of slowing cognitive decline;
- Diclofenac/misoprostol^64^: A non-steroidal anti-inflammatory drug (NSAID) in combination with misoprostol. It did not show clear benefits in the treatment of AD, with the combination not demonstrating efficacy;
- PF-05212377 (SAM-760)^65^: A beta-secretase inhibitor investigated for AD. Clinical results did not show significant positive effects in the treatment of AD;
- Scyllo-Inositol (ELND005)^66^: A compound studied for its potential effect in modulating the formation of amyloid plaques. Trials have not shown substantial clinical benefits;
- Semagacestat^67^: A gamma-secretase inhibitor that has not demonstrated efficacy in delaying cognitive decline and has been associated with adverse effects;
- Huperzine A^68^: A plant-derived acetylcholinesterase inhibitor. Despite some promising initial studies, the evidence was not sufficient to support its large-scale use in AD;
- Tideglusib^69^: An inhibitor of the enzyme glycogen synthase kinase-3 beta (GSK-3β). Studies have not shown significant benefits on cognitive function;
- Sodium selenate^70^: A selenium supplement studied for its potential neuroprotective effect. The results did not indicate clear benefits in AD;
- Capric/Caprylic Acid Triglycerides^71^: These triglycerides are used as a supplement to improve cognitive function. Studies have not provided robust evidence of efficacy for AD;
- Vitamin D2^72^: Although vitamin D plays an important role in general health, studies have not shown that vitamin D2 supplementation has a significant effect on cognitive decline in AD;
- ABT-288^73^: A glutamate receptor antagonist. Studies have not demonstrated clear benefits in AD;
- Simvastatin^74^: A medication used to reduce cholesterol, with some theory about a neuroprotective effect. However, studies have not shown benefits for cognitive decline;
- Atomoxetine^75^: A norepinephrine reuptake inhibitor used for attention deficit hyperactivity disorder (ADHD). Studies have not shown benefits in AD;
- Docosahexaenoic acid^76^: An omega-3 fatty acid studied for its possible neuroprotective effects. The evidence was not sufficient to recommend its use in AD;
- Bexarotene^77^: A retinoid investigated for its ability to reduce amyloid plaques. Studies have not shown clear benefits on cognitive function;
- Hydroxychloroquine^78^: An antimalarial drug studied for its anti-inflammatory effects. Research has not demonstrated significant benefits in AD;
- Levetiracetam^79^: An anticonvulsant investigated for possible neuroprotective effects. Studies have shown no significant impact on cognitive decline;
- Masupirdine^80^: A drug with neuroprotective properties investigated for AD. No clear benefits found in studies;
- Tarenflurbil^81^: An anti-inflammatory investigated for AD, but results did not show significant clinical benefits;
- NeuroAiD™ (MLC601)^82^: A compound supplement investigated for AD, but studies have not shown robust evidence of efficacy;
- Iclepertin (425809)^83^: An N-methyl D-Aspartate (NMDA) receptor modulator, with studies that have not indicated clear benefits in AD;
- Etazolate hydrochloride (EHT0202)^84^: A medication studied for AD, but has not shown significant benefits on cognitive function;
- Vitamin B^85^: Reference to a complex of vitamins from group B. Studies have not shown significant effects on AD;
- MK-7622^86^: A GSK-3β inhibitor investigated for AD but has not demonstrated clinical efficacy;
- Edonerpic maleate (T-817MA)^87^: A compound studied with the expectation of a neuroprotective effect. The results were not positive;
- UB-311^88^: A therapeutic vaccine investigated for AD, but studies have not shown significant benefits;
- Omega-3^89^: Omega-3 fatty acids are often studied for various neurological conditions. However, the evidence for efficacy in AD has not been conclusive;
- Tramiprosate^90^: An anti-amyloid agent investigated for AD but has not shown clear benefits in clinical trials;
- Posiphen^91^: This molecule is an analogue of phenylbutyrate and an inhibitor of amyloid precursor protein (APP). Its main mechanism of action involves reducing the production of the APP protein and, consequently, its metabolites, including amyloid-β peptide (Aβ), which is implicated in the pathogenesis of Alzheimer’s disease (AD). Despite being safe and well tolerated, there were no significant changes in the total ADAS-cog scores.

These examples highlight the complexity and challenges in researching new treatments for Alzheimer’s disease. Most of these medications and supplements have failed to demonstrate a significant impact on disease progression in clinical studies, reflecting the continued need for research to find effective therapeutic options.

### Study limitations

This study has some methodological limitations that may have influenced the results obtained. First, the inclusion and exclusion criteria adopted may have restricted the inclusion of studies that evaluated the use of memantine, a widely used drug approved for the treatment of Alzheimer’s disease, as well as the recently approved lecanemab and donanemab. Although these exclusions were based on previously defined criteria, we recognize that the inclusion of these drugs could provide a more comprehensive view of the available therapeutic options. In addition, the choice to use only the ADAS-Cog scale as the main cognitive outcome may have limited the inclusion of studies that employed other widely recognized measures, such as the Mini-Mental State Examination (MMSE). Although the MMSE is frequently used, its sensitivity and specificity for detecting subtle cognitive changes in moderate to severe stages of Alzheimer’s disease were considered insufficient for the objectives of this review. Finally, although the review was updated until February 2025, new studies and drug approvals continue to emerge. Therefore, the inclusion of more recent data on innovative treatments may be considered in future updates of this study.

In conclusion, medications such as donepezil, galantamine and rivastigmine have demonstrated significant efficacy in improving cognitive function in patients with AD. In turn, other medications have limited or inconclusive evidence: pioglitazone, despite its potential anti-inflammatory effects, has not demonstrated a clear benefit in cognitive improvement, although it may have positive effects in specific subgroups, such as patients with AD and type II diabetes. The combination of doxycycline and rifampicin has shown mixed results, with some evidence of effect, but no definitive conclusions. Metrifonate has shown a positive impact on cognition in some studies, but adverse effects and limited availability make widespread clinical application difficult. Finally, other investigated medications and supplements demonstrated a lack of proven efficacy, such as: acetyl-L-carnitine, turmeric, tulrampator, and scyllo-Inositol, did not show clear benefits in slowing cognitive decline in AD. Study results were inconclusive or did not show statistically significant efficacy. Medications such as semagacestat and bexarotene have also failed to demonstrate a positive impact on cognitive function, and some have been associated with additional adverse effects. Regarding safety and tolerability, among treatments that did not demonstrate significant efficacy, many were well tolerated, with adverse effects that were generally mild and self-limited. However, the lack of consistent efficacy prevents these medications from being recommended for clinical use in AD. Still, the lack of robust evidence for many treatments reflects the need for more research to find effective and safe therapies.

## Data Availability

All data sets were generated or analyzed in the current study.
